# Pituitary Apoplexy: A Retrospective Study of 33 Cases From a Single Center

**DOI:** 10.3389/fendo.2021.656950

**Published:** 2021-04-15

**Authors:** Henrik Falhammar, Sofia Tornvall, Charlotte Höybye

**Affiliations:** ^1^ Department of Endocrinology, Karolinska University Hospital, Stockholm, Sweden; ^2^ Department of Molecular Medicine and Surgery, Karolinska Institutet, Stockholm, Sweden; ^3^ Department of Medicine, Danderyd Hospital, Stockholm, Sweden

**Keywords:** pituitary apoplexy, incidence, symptom, hormone deficiency, management, survival

## Abstract

**Purpose:**

Acute symptomatic pituitary apoplexy is a rare and potentially life-threatening condition. However, pituitary apoplexy can also present with milder symptoms and stable hemodynamics. Due to the rarity of this inhomogeneous condition, clinical studies are important to increase the knowledge.

**Methods:**

We retrospectively reviewed all consecutive cases of pituitary apoplexy being admitted between January 1^st^, 2005 and December 31^st^, 2019 at the Karolinska University Hospital, Stockholm, Sweden, for symptoms, results of magnetic resonance (MRI), biochemistry, management and mortality.

**Results:**

Thirty-three patients were identified with pituitary apoplexy, 18 were men (55%) and mean age was 46.5 (17.2) years. The incidence of symptomatic pituitary apoplexy was 1.6 patients/year (0.76 patients/1,000,000 inhabitants/year). The majority presented with headache (n=27, 82%) and hormonal deficiencies (n=18, 55%), which were most frequent in men. ACTH deficiency was present in nine patients (27% but 50% of those with hormonal deficiencies). All had the characteristic findings on MRI. Only three patients (9%) required acute pituitary surgery, while eight were operated after more than one week. Seven (21%) were on antithrombotic therapy. None of the patients died in the acute course. During follow-up (7.6 ± 4.3 years) none of the hormonal deficiencies regressed and 3 patients died from non-related causes.

**Conclusion:**

Our study confirmed the rarity and the symptoms of this condition. Surprisingly, only 3 patients needed acute neurosurgical intervention, perhaps due to milder cases and a general intensified treatment of precipitating factors. An early awareness and in severe cases decision on pituitary surgery is of utmost importance to avoid severe complications.

## Introduction

Pituitary apoplexy is a rare condition caused by an acute or sub-acute hemorrhage or infarction of the pituitary gland, most often in a pituitary macroadenoma (1). The prevalence and incidence are difficult to estimate as many subacute cases present with subtle symptoms and remain undiagnosed. However, a prevalence of 62 cases/1,000,000 inhabitants (2) and an incidence of 1.7 cases/1,000,000/year (3), have been reported. Previous studies have shown that pituitary apoplexy occurs in 2-12% of patients with pituitary adenoma (1), while hemorrhagic infarction or “subclinical or asymptomatic apoplexy” is more frequent. Up to 25% of all pituitary adenomas display hemorrhagic and/or necrotic areas (4). In 80% a symptomatic apoplexy is the first manifestation of the pituitary adenoma (5). Characteristic symptoms are headache, visual disturbances, nausea and pituitary deficiencies (1, 5). The pathophysiology of pituitary apoplexy is not known, but as pituitary adenomas have less vascularization, ischemia because of tumor growth or decreased blood supply due to an acute blood pressure fall, are among the anticipated explanations (1, 5). The apoplexy generates a specific pattern on magnetic resonance imaging (MRI) of the pituitary with hyper- and hypointense areas in the sella turcica on T1 and T2 weighted images, respectively, suggesting apoplexy (1, 6).

Treatment of acute and severe forms of apoplexy may consist of surgery with decompression and replacement with glucocorticoids, whereas a conservative management may be applied in patients with milder apoplexy without visual and neurological defects (1, 4, 5, 7, 8). However, reports of recovery of neurological, visual and endocrine complications from different managements have varied (7–9).

Thus, there are uncertainties of incidence, management and outcome of pituitary apoplexy. Here we present the results of a review of all patients with confirmed pituitary apoplexy managed in our institution focusing on incidence, presentation, endocrine function, visual deficits, management and mortality in the acute course.

## Materials and Methods

Eligible for inclusion were all consecutive patients with a symptomatic pituitary apoplexy being admitted between January 1^st^, 2005 and December 31^st^, 2019 at the Karolinska University Hospital, Stockholm, Sweden, and where the endocrinologist had been consulted. All the relevant electronic medical files of the patients were reviewed manually up to February 2020. The National Population Register was also consulted to find out if the included patients were still alive and the date of death was retrieved if applicable. The Karolinska University Hospital is a major tertiary hospital (10), with a catchment population for highly specialized care of between 1.9-2.3 million (average 2.1 million) during the study period (https://www.sll.se/globalassets/4.-regional-utveckling/publicerade-dokument/statistik-befolkning-stockhoms-lan-q1-2019.pdf). Approximately 110 patients were diagnosed with a pituitary tumor in Stockholm per year during the study period (https://statistik.incanet.se/Hypofys/Rapport/).

The diagnosis of pituitary apoplexy is mainly clinical, requiring an abrupt or quick onset of specific symptoms as headache, visual or neurological impairment and pituitary deficits. All patients underwent MRI of the sella turcica and were evaluated by a team of experienced neurosurgeons, ophthalmologists, neuroradiologists and endocrinologists. The patients received care in the neurosurgical intensive ward with easy access to other specialists e.g. neurologists, and in close proximity to the radiology department and the operation theater. The patients were continuously monitored for any change in their neurological status or development of other complications. A neurosurgeon experienced in skull base surgery is on call at all times in our hospital. In general, all patients with impaired visual acuity and neurological deficits are operated as soon as possible to improve the outcome.

Pituitary apoplexy does not have a specific International Statistical Classification of Diseases and Related Health Problems (ICD)-code. Therefore, the medical files of all patients referred to our Department during the study period were reviewed for symptoms, MRI and biochemistry. Those, who fulfilled the diagnosis of pituitary apoplexy were included. Two experienced senior endocrinologists (HF and CH) reviewed all cases and only those where agreement on the diagnosis of pituitary apoplexy was reached were included. The age, sex, symptoms, mode of presentation, pituitary characteristics, blood pressure, anticoagulation medications (low molecular heparin, antiplatelet medications, warfarin or novel oral anticoagulants), visual disturbances, biochemical hormonal tests, acute surgery and mortality were recorded. In the acute state ACTH deficiency was based on the patient’s clinical presentation, vital parameters, presence of other pituitary hormone deficiencies and serum cortisol below 200 nmol/L, according to local guidelines. Evaluation with an ACTH test was performed when the situation had stabilized. Blood pressures were measured with an appropriate-sized blood pressure cuff in the Emergency Department or on the ward. All biochemical tests were analyzed according to routine methods.

### Statistical Analysis

Mean (SD) (if normally distributed) or median and range were used. When two groups and continuous variables were compared unpaired t-test (normally distributed) or Mann–Whitney rank-sum test were used as appropriate. Categorical data were reported as numbers with percentages and comparisons were done with Fisher’s exact test. A P-value <0.05 was considered significant.

## Results

Data was collected over the 15-year-period and 33 patients were identified with pituitary apoplexy, of which 24 presented with acute symptoms of pituitary apoplexy. In all patients the diagnosis was supported by characteristic findings on MRI of the pituitary, with hyper- and hypointense areas of different sizes on T1 and T2 weighted images. Thus, the total incidence was 2.2 patients/year (1.04 patients/1,000,000 inhabitants/year) and 1.6 patients/year (0.76 patients/1,000,000 inhabitants/year) for patients with symptomatic pituitary apoplexy. During the same period approximately 1650 patients in Stockholm were diagnosed with a pituitary tumor. Thus, the estimated rate of pituitary apoplexy in pituitary tumors during the study period would be 2% (33/1650).

Of the 33 patients, 18 were men (55%) and 15 women (45%). The mean age was 46.5 (17.2) years (range 15-76 years) and there was a trend towards an older age in men compared to women (51.6 and 46.5 years, respectively, P=0.060) (**Table 1**). Seven patients (21%) were on anticoagulation treatment with acetylsalicylic acid (n=4), clopidogrel (n=1), dalteparin (n=1) and warfarin (n=1). Blood pressure measurements were not available in the emergency situation for all, but for those in whom it was, median systolic blood pressure was 131 mmHg (100–174) and median diastolic blood pressure was 80 mmHg (60-89).

**Table 1:** Characteristics, presentation and surgery of all consecutive cases of pituitary apoplexy admitted at the Karolinska University Hospital during a 15-year-period.

	Total n = 33	Women n = 15	Men n = 18	P-value
Age at onset, mean	46.5 ± 17.2	40.3 ± 13.6	51.6 ± 18.4	0.060
Men, n (%)	18 (55%)			
Acute surgery, n	11 (33%)	3 (20%)	8 (44%)	0.2659
Acute severe, n	24 (73%)	10 (67%)	15 (83%)	0.4184
Anticoagulants, n*	7 (21%)	1 (7%)	6 (35%)	0.0 881
Visual disturbances, n	13 (39%)	3 (20%)	10 (56%)	0.0724
Hormonal deficiency, n**	18 (55%)	3 (21%)	15 (83%)	0.0009
Headache, n	27 (82%)	12 (80%)	15 (83%)	1
Nausea, n	12 (36%)	4 (27%)	8 (44%)	0.4688

*2 were excluded due to missing data.

**1 was excluded due to missing data.

At presentation, headache (n=27, 82%) was the most common symptom. Hormonal deficiencies (n=18, 55%) were most frequent in the men, and TSH and FSH/LH deficiency were observed in most with hormonal deficiencies (**Table 2**). Only one postmenopausal woman had FSH/LH deficiency. Panhypopituitarism was present in 5 patients (males n=4), i.e., 15% of the total cohort and in 26% of males.

**Table 2:** Specification of the hormonal deficiencies encountered in 33 patients with pituitary apoplexy.

Hormonal deficiency, n	All, n = 18	Women, n = 3	Men, n = 15
Panhypopituitarism, n	5 (28%)	1 (33%)	4 (27%)
ACTH, n	9 (50%)	2 (67%)	7 (47%)
TSH, n	12 (67%)	2 (67%)	10 (67%)
FSH/LH, n	15 (83%)	1 (33%)	14 (93%)
GH, n	7 (39%)	1 (33%)	6 (40%)
Prolactin, n	7 (39%)	1 (33%)	6 (40%)

Note that many patients had multiple hormonal deficiencies, thus the total number of deficiencies is larger than the number of patients. The percentages of the individual hormonal deficiencies are from the total number of patients with hormonal deficiencies (i.e., 18, 3 and 15, respectively).

In 28 patients (85%) the pituitary apoplexy was the first manifestation of a non-functioning pituitary adenoma (NFPA), in 4 patients of a macroprolactinoma and in one patient of a Rathke’s pouch cyst. Only 3 patients (9%) underwent acute pituitary surgery, whereas 8 were operated after one week or more. All were operated with endoscopic approach. Two patients were operated because of progressing, pronounced visual defects in addition to oculomotorius nerve palsy and the third patient because of a large, expanding hemorrhage with increasing symptoms of elevated intracerebral pressure and visual defects. No complications to the acute pituitary surgery were noted but the hormonal deficiencies remained unchanged after surgery. The visual defects and oculomotorius nerve palsies all improved greatly but only had a fully visional recovery. All the operated patients had a previously unknown NFPA. None of the patients died in the acute course of the disease. During the 7.6 (4.3) years of follow-up none of the hormonal deficiencies regressed. Three patients (9%) died during follow-up (4-13 years after the pituitary apoplexy), none related to any pituitary causes.

There were three cases with pituitary apoplexy in connection with pregnancy. One female with a macroprolactinoma on dopamine agonist for many years discontinued this medication at gestational week 7. At end of pregnancy (week 37) she presented with nausea, vomiting and headaches. MRI showed a macroadenoma with hemorrhage, i.e., pituitary apoplexy. Glucocorticoids were initiated and she delivered by cesarean section one week later. She continued to have pituitary insufficiency during follow-up and was also restarted on dopamine agonist. A second women was investigated for infertility and was diagnosed with a macroprolactinoma and started on dopamine agonist. After 3 months of dopamine agonist she became pregnant and ceased the medication. At the end of pregnancy, she had an episode of severe headache for a few hours which then spontaneously disappeared. Medical attention was not sought but a few months after delivery MRI showed a recent pituitary hemorrhage. She had no pituitary insufficiencies during follow-up. A third woman had a lot of headache issues during pregnancy. After delivery a bitemporal visual field defect was discovered and a macroadenoma with a recent hemorrhage was diagnosed. Due to the visual field defect she had endoscopic transsphenoidal surgery and the visual field defect recover. She had no pituitary insufficiency during follow-up.

## Discussion

In this study of patients with pituitary apoplexy we found an overall incidence of 1.04 patients/1,000,000 inhabitants/year and 0.76 patients/1,000,000 inhabitants/year of acute symptomatic pituitary apoplexy. The pituitary apoplexy was slightly more frequent in men and presented mostly during their fourth to fifth decade. The most prominent symptom was headache and biochemical levels corresponding to pituitary hormonal deficiency. In the present cohort conservative management worked well in the majority of patients.

The diagnosis of pituitary apoplexy can be difficult, unless the patient present with characteristic symptoms that are acute (1, 4, 5). Especially subacute apoplexy can be assumed to be under-diagnosed due to unspecific symptoms. In our study the rate of pituitary apoplexy was lower than previously reported (3). One explanation might be that not all patients were referred to our hospital, but this is unlikely since the hospital is the tertiary reference hospital and the only hospital with a neurosurgical department in the region. All patients diagnosed with pituitary apoplexy can therefore be anticipated to have been referred to our hospital and the endocrinologist consequently consulted. Another explanation could be related to differences in the definition of acute pituitary apoplexy and the severity of it compared to the definition used in other studies. In a previous study, a four times higher prevalence of pituitary apoplexy than earlier studies was reported, but data was retrieved from different general practice surgeries with a population of only 81,449 inhabitants (2). In a Finnish study, the incidence of pituitary apoplexy was 1.7 cases/1,000,000 inhabitants/year (3), which was a little higher than our total incidence of 1.04 patients/1,000,000 inhabitants/year. Moreover, the last decades there has also been a dramatic increase of CT and MRIs performed where the pituitary gland can be visualized (11), and thus more subclinical or asymptomatic pituitary apoplexy may be diagnosed. The CT is usually most valuable in the first 48 hours after the apoplexy, whereafter MRI is better in detecting a pituitary adenoma and its haemorrhagic degeneration as the blood degradation decreases the density on CT (1). We tried not to over-diagnose pituitary apoplexy, i.e., we did not include patients with signs on MRI of hemorrhagic degradation areas in previously known pituitary tumors and without symptoms consistent with pituitary apoplexy. During the present study, it became evident that even the acute and severe cases can be difficult to diagnose. Some were misinterpreted initially as e.g. meningitis, stroke and even gastroenteritis. In addition, the fact that not all minor hospitals have access to MRI, and certainly not acute MRI, may contribute to a lower incidence.

Another finding in our study was that the male to female ratio was not so pronounced. In a review of 25 different studies the male to female ratio varied between the studies, with generally higher ratios in the earlier studies, but the average was 1.4 (1), which is similar to our ratio of 1.2. Over time treatment of hypertension and hypercholesterolemia in general has intensified, which might have had a positive effect in reducing the risk of pituitary apoplexy and possibly reduced the male to female ratio.

Characteristic symptoms of pituitary apoplexy are headache, which we found in 82% of our patients, visual disturbances in 39% and nausea in 36%, which is similar to previous studies (1, 5). Endocrine deficiencies were seen in 55% of our patients and have been reported previously up to 85% of cases but with an average of 64% (1, 9). In the present study most patients with hormonal deficiency had TSH and/or gonadotrophin deficiencies while 50% had ACTH deficiency but only 39% GH deficiency. In contrast, previous studies have found that those diagnosed with hormonal deficiency have almost all had GH deficiency, while ACTH deficiency was reported in 50-80%, TSH deficiency in 30-70% and FSH/LH deficiency in 40-75% (1). The lower frequency of GH deficiency can depend on how these were diagnosed, in the acute situation only IGF-I levels were evaluated to diagnose GH deficiency and it is well-known that IGF-I levels may underestimate GH deficiency (12). Evaluation for GH deficiency was performed later with GH stimulation test, according to international guidelines. Also, prolactin deficiency and diabetes insipidus have been observed in some cases but we found a quite high frequency of prolactin deficiency (7/33 cases, 21%) and panhypopituitarism (15%) suggesting that these patients had severe pituitary apoplexy. The most important hormone insufficiency in the acute state is the ACTH deficiency, which may be life-threatening if not replaced (13). In this context, it should be noted that it can be very difficult to separate symptoms and signs of an acute apoplexy from symptoms and signs of ACTH deficiency. The observed differences in pituitary function could be due to differences in diagnosing hormonal deficiency, in particular during the acute course. However, pituitary deficiencies, once established, usually do not recover (14). Of interest, one study showed a better endocrinologic outcome in patients with pituitary apoplexy who underwent endoscopic pituitary surgery compared to patients who were treated conservatively (15). However, our patients with pituitary deficiencies did not recover irrespective whether they had endoscopic pituitary surgery or not.

The first treatment intervention in patients with pituitary apoplexy is hemodynamic stabilization, correction of electrolyte levels and treatment with glucocorticoids (16). Most patients with pituitary apoplexy improve with either surgical or conventional treatment but the best approach and the timing of surgery is controversial (16–18). In patients with milder symptoms and an improving course of the disease a conservative approach is usually sufficient under continued ophthalmological, neurological and endocrine monitoring (19). On the other hand, it has been shown that pituitary surgery within 7 days of the apoplexy leads to better outcome for visual defects (4, 16, 18). It is important that the treatment is adjusted to the patient’s symptoms and the course of the disease. In accordance with many studies, the majority of patients in our study could be managed conservatively and only 3 patients (9%) required acute surgical intervention (1, 5, 7, 8). Acute pituitary apoplexy initially requires careful and repeated physical examinations as the patient’s condition may worsen rapidly. Intracranial hypertension and diencephalic alterations due to direct compression with stupor or coma, or arterial occlusion due to vasospasm or direct compression with stroke might develop. Immediate or early neurosurgery is always indicated in patients with progressive ophthalmological or neurological impairments (19). The decision for conservative or neurosurgical intervention should be done by a multidisciplinary team and based on the clinical status of the patient together with the result of focused sellar imaging (4). An experienced pituitary neurosurgeon is preferred since this decreases the risk of adverse events. As a guiding tool for deciding on surgical management, algorithms can be helpful. The UK guidelines contain a scoring system, that includes visual acuity, visual defects, cranial neve palsies and Glasgow Coma Scale (4). Scores range from 0 to 10 and a score above 4 suggests surgery (4). Another grading system to classify the clinical spectrum of pituitary apoplexy uses a scale from 1 to 5. Grade 1 patients are asymptomatic with radiographic pituitary apoplexy(“subclinical” apoplexy), grade 2 patients have endocrinopathy with appropriate radiographic findings but no other clinical symptoms, grade 3 patients have headache, grade 4 patients have ocular paresis, and grade 5 patients have acute visual deficits or decreased Glasgow Coma Scale score preventing accurate visual assessment (20). According to this scoring system, patients who receive grade 5 or higher should be operated. In the present cohort 13 patients had visual disturbances. Eleven of these improved during conservative treatment but progressed in two, and these two patients together with one patient with severe neurological symptoms were acutely operated. These patients would all receive grade 5.

A minority of patients with pituitary apoplexy will have precipitating factors (16). The most common precipitating factors are pituitary stimulation, surgery (in particular coronary artery surgery), and coagulopathy (16). Thus, antithrombotic/anticoagulant therapy may have an important role as precipitating factor (15), and in the current study 21% of the patients were on such treatment. Over time the new, easier controllable anticoagulants that have become in use probably also decrease the number of patients at risk of a pituitary hemorrhage. High blood pressure is another well-known risk factor for pituitary apoplexy, but in the present study this was not noted.

Gestational pituitary apoplexy is a rare event. Women with prolactinomas who became pregnant soon after starting treatment with dopamine agonist and the rapid withdrawal following pregnancy confirmation can lead to pituitary apoplexy and serious neurological symptoms as well as secondary adrenal insufficiency (21). Pituitary surgery may then be necessary even during pregnancy after careful consideration. In the present study, three women had pituitary apoplexy in connection with pregnancy, two after withdrawal of dopamine agonist, and one needed pituitary surgery after delivery due to visual field defects (nonfunctioning macroadenoma). Thus, in pregnant women with a pituitary tumor and symptoms of pituitary apoplexy and urgent MRI should be considered.

This study has some limitations like all retrospective studies including missing data. The systematic review of all cases of pituitary apoplexy in our institution may have been incomplete due to not being identified. Moreover, not all cases may have been admitted to our tertiary center but may have been admitted to other secondary hospitals. Furthermore, even though this study had a decent amount of patients included compared to most previous studies of pituitary apoplexy (1), the number of patients was still limited. Due to these limitations, the results, particularly about the ideal management of pituitary apoplexy, should be interpreted with caution. However, it is difficult in such a rare condition to plan a single center prospective study with sufficient number of participants.

## Conclusion

In summary, our study confirmed a low incidence of acute pituitary apoplexy with a slight male predominance. The main symptom was headache and the most common acute pituitary hormonal deficiencies were TSH and FSH/LH deficiency. However, 50% of those with a hormonal deficiency had ACTH deficiency which itself is life-threatening if not replaced. Large, expanding pituitary bleedings in need of acute surgical intervention were very rare, maybe indicating increased access to MRI, intensified treatment of hypertension and use of easier controllable anticoagulation therapy and thereby over time decreasing the number of patients at risk. To conclude, symptoms are unspecific, and an early awareness of pituitary apoplexy and decision for pituitary surgery is still of utmost importance to avoid severe complications.

## Data Availability Statement

The raw data supporting the conclusions of this article will be made available by the authors, without undue reservation.

## Ethics Statement

The studies involving human participants were reviewed and approved by Swedish Ethical Review Authority. Written informed consent for participation was not required for this study in accordance with the national legislation and the institutional requirements.

## Author Contributions

All authors (HF, ST and CH) were involved in the study design, statistical analysis, interpreting data, and manuscript drafting. All authors contributed to the article and approved the submitted version.

## Funding

This was an academic investigation with funding from the Magnus Bergvall Foundation.

## Conflict of Interest

The authors declare that the research was conducted in the absence of any commercial or financial relationships that could be construed as a potential conflict of interest.

## References

[1] Briet C Salenave S Bonneville JF Laws ER Chanson P. Pituitary Apoplexy. Endocr Rev (2015) 36(6):622–45. 10.1210/er.2015-1042 26414232

[2] Fernandez A Karavitaki N Wass JA. Prevalence of pituitary adenomas: a community-based, cross-sectional study in Banbury (Oxfordshire, UK). Clin Endocrinol (Oxf) 72(3):377–82. 10.1111/j.1365-2265.2009.03667.x 19650784

[3] Raappana A Koivukangas J Ebeling T Pirila T. Incidence of pituitary adenomas in Northern Finland in 1992-2007. J Clin Endocrinol Metab 95(9):4268–75. 10.1210/jc.2010-0537 20534753

[4] Rajasekaran S Vanderpump M Baldeweg S Drake W Reddy N Lanyon M. UK guidelines for the management of pituitary apoplexy. Clin Endocrinol (Oxf) 74(1):9–20. 10.1111/j.1365-2265.2010.03913.x 21044119

[5] Biousse V Newman NJ Oyesiku NM. Precipitating factors in pituitary apoplexy. J Neurol Neurosurg Psychiatry 71(4):542–5. 10.1136/jnnp.71.4.542 PMC1763528 11561045

[6] Chapman PR Singhal A Gaddamanugu S Prattipati V. Neuroimaging of the Pituitary Gland: Practical Anatomy and Pathology. Radiol Clin North Am 58(6):1115–33. 10.1016/j.rcl.2020.07.009 33040852

[7] Almeida JP Sanchez MM Karekezi C Warsi N Fernandez-Gajardo R Panwar J. Pituitary Apoplexy: Results of Surgical and Conservative Management Clinical Series and Review of the Literature. World Neurosurg (2019) 130:e988–99. 10.1016/j.wneu.2019.07.055 31302273

[8] Barkhoudarian G Kelly DF. Pituitary Apoplexy. Neurosurg Clin N Am (2019) 30(4):457–63. 10.1016/j.nec.2019.06.001 31471052

[9] Seo Y Kim YH Dho YS Kim JH Kim JW Park CK. The Outcomes of Pituitary Apoplexy with Conservative Treatment: Experiences at a Single Institution. World Neurosurg (2018) 115:e703–10. 10.1016/j.wneu.2018.04.139 29709755

[10] Falhammar H Wallin G Calissendorff J. Acute suppurative thyroiditis with thyroid abscess in adults: clinical presentation, treatment and outcomes. BMC Endocr Disord 19(1):130. 10.1186/s12902-019-0458-0 PMC6889346 31791298

[11] Crowther S Rushworth RL Rankin W Falhammar H Phillips LK Torpy DJ. Trends in surgery, hospital admissions and imaging for pituitary adenomas in Australia. Endocrine 59(2):373–82. 10.1007/s12020-017-1457-4 29103185

[12] Molitch ME Clemmons DR Malozowski S Merriam GR Vance ML Endocrine S. Evaluation and treatment of adult growth hormone deficiency: an Endocrine Society clinical practice guideline. J Clin Endocrinol Metab 96(6):1587–609. 10.1210/jc.2011-0179 21602453

[13] Rushworth RL Torpy DJ Falhammar H. Adrenal Crisis. N Engl J Med (2019) 381(9):852–61. 10.1056/NEJMra1807486 31461595

[14] Glezer A Bronstein MD. Pituitary apoplexy: pathophysiology, diagnosis and management. Arch Endocrinol Metab (2015) 59(3):259–64. 10.1590/2359-3997000000047 26154095

[15] Teixeira JC Lavrador J Simão D Miguéns J. Pituitary Apoplexy: Should Endoscopic Surgery Be the Gold Standard? World Neurosurg (2018) 111:e495–9. 10.1016/j.wneu.2017.12.103 29288106

[16] Wildemberg LE Glezer A Bronstein MD Gadelha MR. Apoplexy in nonfunctioning pituitary adenomas. Pituitary (2018) 21(2):138–44. 10.1007/s11102-018-0870-x 29383476

[17] Bi WL Dunn IF Laws ER Jr. Pituitary apoplexy. Endocrine (2020) 48(1):69–75. 10.1007/s12020-014-0359-y 25063308

[18] Abdulbaki A Kanaan I. The impact of surgical timing on visual outcome in pituitary apoplexy: Literature review and case illustration. Surg Neurol Int (2017) 8:16. 10.4103/2152-7806.199557 28217395 PMC5309450

[19] Giammattei L Mantovani G Carrabba G Ferrero S Di Cristofori A Verrua E. Pituitary apoplexy: considerations on a single center experience and review of the literature. J Endocrinol Invest (2016) 39(7):739–46. 10.1007/s40618-015-0424-2 26733212

[20] Jho DH Biller BM Agarwalla PK Swearingen B. Pituitary apoplexy: large surgical series with grading system. World Neurosurg (2014) 82(5):781–90. 10.1016/j.wneu.2014.06.005 24915069

[21] Witek P Zieliński G Maksymowicz M Zgliczyński W. Transsphenoidal surgery for a life-threatening prolactinoma apoplexy during pregnancy. Neuroendocrinol Lett (2012) 33(5):483–8. 23090264

